# Postoperative Pelvic Abscess Risk in Gynecologic Surgery: A Four-Year Single Institution Experience

**DOI:** 10.7759/cureus.94383

**Published:** 2025-10-12

**Authors:** Dwight Im

**Affiliations:** 1 Gynecologic Oncology Center, Mercy Medical Center, Baltimore, USA

**Keywords:** complications, gynecologic oncologic surgery, gynecologic surgery, hemostatic agents, pelvic abscess, surgicel, tisseel

## Abstract

Background

Hemostatic agents (HAs) are often used in gynecologic surgery to control bleeding when conventional methods such as sutures, clips, and electrocautery are insufficient. Tisseel Fibrin Sealant (FS) and oxidized regenerated cellulose (ORC) (e.g., Surgicel Powder) are two HAs that are commonly utilized; however, there is limited clinical data regarding their safety profiles, specifically regarding the risk of postoperative complications such as pelvic abscess. This study was initiated following observations of increased readmissions for pelvic abscesses in patients receiving ORC.

Objective

The primary objective of this retrospective study was to evaluate the association between the intraoperative use of FS versus ORC and the incidence of postoperative pelvic abscess and other associated complications in patients undergoing gynecologic surgery.

Methods

A retrospective chart review was conducted on women who underwent gynecologic surgery by the author at a single academic institution in Baltimore, Maryland, between 2019 and 2023. Patient and surgical characteristics were extracted, and comparisons were made between those who received FS versus ORC. The association between intraoperative HA use and postoperative pelvic abscess formation was examined. Secondary outcomes included the assessment of other major postoperative complications.

Results

A total of 607 patients were identified, with 422 receiving ORC and 185 receiving FS; 72 (11.9%) patients experienced postoperative complications. Pelvic abscess occurred in 10.4% (95% CI, 7.5%-13.3%) of ORC-treated patients compared with 0% (95% CI, 0%-1.6%) of FS-treated patients (p < 0.05). The overall complication rate was significantly greater at 15.6% (95% CI, 12.2-19.0%) for ORC versus 3.2% (95% CI 0.7%-5.8%) for FS. Readmission within 30 days of surgery occurred in 12.1% (95% CI, 9.0%-15.2%) of ORC-treated patients compared with 0.5% (95% CI, 0%-1.6%) of FS-treated patients, a significantly higher rate among those receiving ORC.

Conclusion

The use of ORC for adjunctive hemostasis in gynecologic surgery between 2019 and 2023 was associated with a significantly higher risk of pelvic abscess and other postoperative complications compared to FS. These findings suggest that FS may be a safer alternative for managing bleeding in gynecologic procedures, particularly in patients at higher risk for postoperative infections. Enhanced regimens of antibiotic prophylaxis are clinically significant and should be considered when ORC is used. These findings warrant prospective studies to validate these results and optimize clinical practice.

## Introduction

During open and minimally invasive gynecologic surgery, the main techniques used to achieve hemostasis are sutures, hemoclips, and electrocautery [1]. However, in situations where these methods are inadequate, various hemostatic agents (HAs) can be utilized [1]. These agents include mechanical, passive agents such as oxidized regenerated cellulose (ORC) (e.g., Surgicel Powder), as well as active biologic agents containing thrombin and fibrin, such as Tisseel Fibrin Sealant (FS) [2]. Despite the increasing use of HAs, there is limited comparative data available in gynecologic surgery.

The composition of ORC is a plant-based polymer consisting of fibers with mechanical hemostatic properties [3-5]. The mechanisms by which ORC facilitates hemostasis are not completely understood [2,6]; however, preclinical studies indicate that the low pH may delay wound healing and create inflammation [7].

As a two-component, active HA, FS consists of fibrinogen and thrombin that exploit the final stage of the coagulation cascade [8]. A clot is formed by FS that closely mimics the natural physiological process and also includes a clot stabilizer (aprotinin) that ensures clot integrity during the critical phases of wound healing [8].

In 2021, the author noted a trend in readmissions for pelvic abscess that seemed to be correlated with the use of ORC. Therefore, he discontinued ORC use, only utilizing FS for adjunctive hemostasis, and noted an anecdotal reduction in abscess formation. Few studies have evaluated the relationship between HAs and pelvic abscess formation. Therefore, this study reports results from the author’s retrospective assessment of perioperative safety and efficacy following ORC and FS administration in his patients, with the hypothesis that FS would be associated with a lower incidence of postoperative infections compared to ORC.

## Materials and methods

A retrospective chart review was performed at Mercy Medical Center and met the criteria for exempt review by the Institutional Review Board of this facility. The study population included adult females aged 18 to 95 years. To meet the inclusion criteria, patients were required to have undergone gynecologic surgery between 2019 and 2023, received ORC or FS for adjunctive hemostasis, and had sufficient follow-up data for evaluation. Patients were excluded if they did not receive ORC or FS and lacked sufficient follow-up data for evaluation.

The primary objective of this retrospective study was to evaluate the association between the intraoperative use of FS versus ORC and the incidence of postoperative pelvic abscess and other associated complications in patients undergoing gynecologic surgery. The primary hypothesis was that the use of FS would result in a lower incidence of postoperative infections when compared to the ORC.

All patient data were de-identified. Demographic data, including date of surgery, age, height, weight, and body mass index (BMI), were documented. In addition, relevant past medical history, comorbidities, and concomitant medications, including preoperative antibiotics, were evaluated. Race and ethnicity data were not gathered during the data review process; the author acknowledges that the lack of such data may potentially confound the findings and that the retrospective nature of the study introduces inherent biases. Laboratory values, including white blood cells (WBCs) and hemoglobin (Hgb), were also assessed. Diagnostic and surgical data included primary diagnosis, postoperative diagnosis, surgical procedure/intraoperative data, and length of stay. Safety data were categorized based on the HA utilized (i.e., ORC or FS) and included all postoperative complications and readmissions.

The primary outcome of interest was the development of pelvic abscess, which was defined as an organized pelvic fluid collection with associated systemic signs of infection, identified in the medical record within 30 days of surgery. Descriptive statistics were used to compare the variables between groups. Hypothesis testing was carried out using a t-test. A p-value of ≤ 0.05 was considered statistically significant. Ninety-five percent confidence intervals (95% CIs) for proportions were calculated using the Wilson score method. All analyses were conducted using SAS version 9.4 (SAS Institute Inc., Cary, NC).

## Results

Patient characteristics

A total of 607 patients were included in the study, with 422 receiving ORC and 185 receiving FS (Table 1). The median age of the patients in the ORC group was 54 years (range 23-92 years) and 56 years (24-92 years) in the FS group, indicating no significant difference between the groups. The mean BMI was 33.2 kg/m² (SD = 9.5 kg/m²), with no significant difference between the groups. The majority of patients were within the age range of 40-60 years, and there was a comparable distribution of height and weight across both groups.

**Table 1:** Demographics and baseline characteristics BMI = body mass index; FS = fibrin sealant; Hgb = hemoglobin; NS = not significant; ORC = oxidized regenerated cellulose; SD = standard deviation; WBC = white blood cell *Please interpret any result with a p-value <0.05 compared to 0 (0.0) with caution.

Patient Characteristics	Total Patients	ORC	FS	p-value*
N	607	422	185	
%	100%	69.5%	30.5%	
Age (years), median	54	54	56	NS
Age (years), (min, max)	(23, 92)	(23, 92)	(24, 92)	-
Height (cm), mean (SD)	163.7 (7.3)	163.6 (7.2)	163.7 (7.4)	NS
Weight (kg), mean (SD)	89.5 (27.3)	89.9 (27.3)	88.5 (27.4)	NS
BMI (kg/m^2^), mean (SD)	33.2 (9.5)	33.4 (9.4)	32.9 (9.7)	NS
Laboratory values (preoperative)				
WBC, n (%)	560 (92.3)	397 (94.1)	183 (98.9)	NS
WBC (10^9^/L), mean (SD)	6.95 (2.23)	6.96 (2.25)	6.92 (2.19)	NS
Hgb (g/dL), n (%)	560 (92.3)	397 (94.1)	183 (98.9)	NS
Hgb (g/dL), mean (SD)	12.62 (1.67)	12.56 (1.72)	12.74 (1.57)	NS
List of preoperative antibiotics, n (%)				
Cefazolin	386 (63.6)	348 (82.5)	38 (20.5)	<0.05
Cefazolin, metronidazole	154 (25.4)	20 (4.7)	134 (72.4)	<0.05
Clindamycin	33 (5.4)	33 (7.8)	0 (0.0)	<0.05
Clindamycin, gentamicin	13 (2.1)	9 (2.1)	4 (2.2)	NS
Clindamycin, metronidazole	4 (0.7)	0 (0.0)	4 (2.2)	<0.05
Metronidazole	4 (0.7)	1 (0.2)	3 (1.6)	<0.05
None	3 (0.5)	3 (0.7)	0 (0.0)	<0.05
Cefotetan	2 (0.3)	1 (0.2)	1 (0.5)	NS
Cefotetan, metronidazole	2 (0.3)	2 (0.5)	0 (0.0)	<0.05
Gentamicin, metronidazole	2 (0.3)	1 (0.2)	1 (0.5)	NS
Clindamycin, gentamicin, metronidazole	1 (0.2)	1 (0.2)	0 (0.0)	<0.05
Gentamicin	1 (0.2)	1 (0.2)	0 (0.0)	<0.05
Vancomycin	1 (0.2)	1 (0.2)	0 (0.0)	<0.05
Cefazolin, cefotetan	1 (0.2)	1 (0.2)	0 (0.0)	<0.05

Preoperative laboratory values, including WBC count and Hgb, showed no significant differences between the groups, indicating comparable baseline health status. The majority of patients received preoperative antibiotics, with Cefazolin being the most common (63.6%). A significant difference was observed in the use of combination antibiotics, with 72.4% of patients in the FS group receiving Cefazolin and Metronidazole compared with 4.7% in the ORC group. This suggests a tailored approach to antibiotic prophylaxis based on the type of HA used. The detailed list of preoperative antibiotics is provided in Table 1, highlighting the variations in antibiotic regimens and their potential impact on postoperative outcomes.

Surgical characteristics

Surgical characteristics are detailed in Table 2. The majority of surgeries were robotic-assisted, with a higher proportion in the FS group compared with the ORC group. This statistically significant difference suggests a preference for using FS in minimally invasive procedures. The distribution of surgeries performed each year showed a significant variation, particularly in 2022 and 2023, with a higher number of FS cases compared with ORC, reflecting the shift in clinical practice by the author over time. The mean duration of surgery was 106.1 minutes (SD = 32.9 minutes), with the ORC group having a slightly shorter average duration compared with the FS group, though this difference was not statistically significant. The length of hospital stay was significantly shorter for patients in the FS group (mean = 29.2 hours, SD = 22.6 hours) compared with the ORC group (mean = 43.5 hours, SD = 73.6 hours), suggesting that FS may contribute to faster postoperative recovery.

**Table 2:** Surgical characteristics FS = fibrin sealant; Hgb = hemoglobin; NS = not significant; ORC = oxidized regenerated cellulose; SD = standard deviation; WBC = white blood cell *Patients with robotic surgery were all labeled as laparoscopic type; percentage calculated with non-robotic assisted as the denominator. **Please interpret any result with a p-value <0.05 compared to 0 (0.0) with caution.

Surgery Characteristics	Total Patients	ORC	FS	p-value**
N	607	422	185	
%	100%	69.5%	30.5%	
Type of surgery, n (%)				
Robotic-assisted*	539 (88.8)	367 (86.9)	172 (93.0)	<0.05
Non-robotic assisted	68 (11.2)	55 (13.0)	13 (7.0)	<0.05
Laparotomy*	61 (89.7)	50 (90.9)	11 (84.6)	-
Laparoscopic*	7 (10.3)	5 (9.1)	2 (15.4)	-
Year surgery was performed, n (%)				
2019	121 (19.9)	121 (28.7)	0 (0.0)	<0.05
2020	180 (29.7)	180 (42.7)	0 (0.0)	<0.05
2021	121 (19.9)	118 (28.0)	3 (1.6)	<0.05
2022	100 (16.5)	1 (0.2)	99 (53.5)	<0.05
2023	85 (14.0)	2 (0.5)	83 (44.9)	<0.05
Surgery time				
Duration of surgery (minutes), mean (SD)	106.1 (32.9)	104.8 (33.4)	109.08 (31.7)	NS
Duration of surgery (minutes), (min, max)	(40, 390)	(40, 390)	(40, 290)	-
Length of hospital stay (hours), mean (SD)	39.2 (62.9)	43.5 (73.6)	29.2 (22.6)	<0.05
Surgery outcomes (postoperative day 1), n (%)				
Complications within 30 days from surgery	72 (11.9)	66 (15.6)	6 (3.2)	<0.05
Readmission within 30 days from surgery	52 (8.6)	51 (12.1)	1 (0.5)	<0.05
(Postoperative day 1)				
WBC, n (%)	541 (89.1)	379 (89.8)	162 (87.6)	NS
WBC (10^9^/L), mean (SD)	10.89 (3.05)	10.99 (2.97)	10.67 (3.24)	NS
Hgb (g/dL), n (%)	541 (89.1)	379 (89.8)	162 (87.6)	NS
Hgb (g/dL), mean (SD)	10.95 (1.58)	10.95 (1.57)	10.97 (1.58)	NS
Change from baseline (preoperative), mean (SD)				
Change in WBC from baseline (increase)	3.94 (0.82)	4.03 (0.72)	3.75 (1.05)	NS
Change in Hgb from baseline (decrease)	1.67 (0.09)	1.61 (0.15)	1.77 (0.01)	NS

Postoperative complications

A total of 72 patients (11.9%) experienced postoperative complications, with a significantly higher incidence in the ORC group (15.6%; 95% CI, 12.2%-19.9%) compared with the FS group (3.2%; 95% CI, 0.7%-5.8%; Table 3 and Table 4). The most common complication was pelvic abscess, occurring in 61.1% of patients with complications (Table 4). Notably, all pelvic abscesses were in the ORC group (10.4%; 95% CI, 7.5%-13.3%), with none reported in the FS group (0%; 95% CI, 0%-1.6%). This statistically significant finding underscores the association between ORC and the risk of abscess formation. Other complications included acute kidney injury, ileus, and sepsis, all significantly higher in the ORC group. Complications occurring in less than 4 patients were relatively common postoperative complications such as pain, urinary retention, and seroma, and all resolved within the immediate postoperative period. However, the preponderance of complications occurred in the patients receiving ORC.

**Table 3:** Overall summary of complication data

	Total Patients in Study N = 607 (100%)		
	Patients with complications (N = 72)	Patients without complications (N = 535)	Total N
Oxidized regenerated cellulose	66 (15.6%)	356 (84.4%)	422 (69.5%)
Fibrin sealant	6 (3.2%)	179 (96.8%)	185 (30.5%)
Total N	72 (11.9%)	535 (88.1%)	607 (100%)

**Table 4:** Complications in >3 patients FS = fibrin sealant; ORC = oxidized regenerated cellulose. *Not mutually exclusive; one patient can have multiple complications. **Please interpret any result with a p-value <0.05 compared to 0 (0.0) with caution.

Surgical Complications	Total Patients	ORC	FS	p-value**
N	72	66	6	
Types of complications, n (%)*				
Pelvic abscess	44 (61.1)	44 (66.7)	0 (0.0)	<0.05
Acute kidney injury	7 (9.7)	6 (9.1)	1 (16.7)	<0.05
Ileus	5 (6.9)	5 (7.6)	0 (0.0)	<0.05
Sepsis	4 (5.6)	4 (6.1)	0 (0.0)	<0.05

The data suggest that the acidic pH of ORC may contribute to an inflammatory response, promoting granuloma formation and subsequent abscess development. This hypothesis is supported by findings from Fagotti and colleagues [9], who postulated similar mechanisms. The higher rate of complications in the ORC group suggests a need for careful consideration when selecting HAs, particularly in patients with higher risk profiles.

Readmissions

The readmission rate within 30 days of surgery was significantly higher in the ORC group (12.1%; 95% CI, 9.0%-15.2%) compared with the FS group (0.5%; 95% CI, 0%-1.6%) (Table 2). This finding is critical as it reflects the broader impact of the choice of HA on healthcare resource utilization and patient quality of care. The high readmission rate associated with ORC highlights the need for more effective preoperative and postoperative management strategies to mitigate these risks if this product is utilized.

Antibiotic subanalysis

A subanalysis was conducted to determine the antibiotic regimen utilized in the patients who experienced complications of pelvic abscess and sepsis (all patients receiving ORC; Table 5). An important finding from the antibiotic subanalysis demonstrated that the use of combination preoperative antibiotics was associated with a significant reduction in the rate of pelvic abscess in patients who received ORC. Specifically, the combination of cefazolin and metronidazole usage was associated with a significantly lower incidence (4.5%; 95% CI, 0.8%-16.5%) of pelvic abscess compared with patients receiving cefazolin alone (63.6%; 95% CI, 48-8%-76.4%). Patients receiving a combination of cefazolin and cefotetan had an even lower incidence (2.3%; 95% CI, 0.4%-12.0%) of pelvic abscess. This suggests that enhanced antibiotic prophylaxis can mitigate some of the risks associated with ORC use. This subanalysis underscores the importance of tailored antibiotic strategies to improve surgical outcomes. However, it is noted that the relatively small sample size may reduce the power to detect smaller differences in outcomes between the subgroups.

**Table 5:** Preoperative antibiotics utilized in oxidized regenerated cellulose (ORC) patients with complications of pelvic abscess and sepsis

	ORC Patients with Complications (N = 66)	
	Pelvic abscess	Sepsis
N	44 (66.7)	4 (6.1)
List of preoperative antibiotics, n (%)		
Cefazolin	28 (63.6)	4 (100.0)
Cefazolin, cefotetan	1 (2.3)	0 (0.0)
Cefazolin, metronidazole	2 (4.5)	0 (0.0)
Clindamycin	10 (22.7)	0 (0.0)
Clindamycin, gentamicin	2 (4.5)	0 (0.0)
Metronidazole	1 (2.3)	0 (0.0)

Comparison with the literature

The findings of this study align with previous research, such as the study by Fagotti and colleagues [9], which also reported a higher incidence of postoperative abscesses associated with ORC. Furthermore, the systematic review by Masoudi and colleagues [6] supports the broad spectrum of complications that can arise from using ORC products, including granulomas and misdiagnosed masses. These complications underscore the need for careful selection and management of HAs in surgical practice. Additional studies highlight the potential for misdiagnosis due to the radiographic appearance of ORC, further complicating postoperative management [10].

Clinical implications

The use of FS in this study was associated with a notable absence of pelvic abscesses, highlighting its potential benefits over ORC for adjunctive hemostasis in gynecologic surgery. The mechanism of action of FS involves forming a stable fibrin clot that mimics natural coagulation processes, which may explain its superior safety profile [8]. Given the significant differences in postoperative outcomes between ORC and FS, these findings have important clinical implications for the selection of HAs in gynecologic surgeries. Surgeons should weigh the benefits and risks of each agent and consider enhanced antibiotic prophylaxis strategies, especially when using ORC.

Summary of complications

Overall, patients in the ORC group had a significantly higher rate of complications, readmissions, and postoperative infections compared with those in the FS group. The complications experienced in greater than three patients, as detailed in Table 4, provide a clear picture of the risks associated with each HA. The overall summary of complication data, presented in Table 3, underscores the importance of careful agent selection and effective preoperative and postoperative management to improve patient outcomes.

## Discussion

This retrospective study found a significant association between the use of ORC and the subsequent formation of pelvic abscesses in gynecologic surgery, with a higher incidence of sepsis compared to FS. Notably, all pelvic abscesses occurred in patients who received ORC, with none reported in the FS group. These findings suggest that the use of ORC in gynecologic procedures should be carefully considered, especially in patients with higher risk profiles or those undergoing extensive surgical interventions.

The results of this study align with previous research. A study by Fagotti and colleagues [9] found that the use of ORC was significantly associated with postoperative abscesses in gynecologic oncology surgeries. These data suggest that the type of HAs used can influence the incidence of postoperative complications.

While the mechanism by which ORC is believed to achieve hemostasis is not entirely clear, it potentially occurs due to multiple mechanisms, including activation of the intrinsic coagulation pathway, creation of a low pH environment to promote vasoconstriction, and formation of a distinct scaffold for establishing a platelet plug [11]. Fagotti and colleagues [9] postulated that the acidic pH of ORC may support a proinflammatory response promoting the formation of a granuloma or foreign body reaction.

A recent systematic review by Masoudi and colleagues [6] provided detailed data on complications that may be associated with ORC products, including masses (granulomas, abscesses, hematomas, cysts), hemorrhagic complications, misdiagnosed masses, cardiovascular, nervous system, and hepatobiliary complications, pain, and infections. Other complications included fistulas, erectile dysfunction, chorioamnionitis, swelling, urinary leak, renal failure, and anaphylaxis. This broad spectrum of potential complications underscores the need for careful consideration when choosing HAs.

Tam and colleagues [10] described cases where ORC was mistaken for abscesses on imaging studies, leading to potential misdiagnoses and unnecessary treatments. Behbehani and Tulandi [12] reported similar findings, where ORC mimicked abscess formation in the postoperative period, complicating clinical management. These findings further validate the need for heightened awareness and precise clinical decision-making.

In another gynecologic surgery study in patients undergoing hysterectomy, the risk of pelvic abscess formation following the use of ORC, or a flowable matrix (Surgiflo Matrix), in a small minority of patients, was evaluated [13]. The results demonstrated that the use of these agents was associated with an increase in 30-day presentation to the Emergency Department and readmission. However, the study was insufficiently powered to find a statistically significant difference in postoperative abscess formation with the use of HAs.

An important aspect of this study was the antibiotic subanalysis. The use of a combination of preoperative antibiotics significantly reduced the rate of pelvic abscess in patients who received ORC. This finding suggests that enhanced antibiotic prophylaxis can mitigate some of the risks associated with the use of ORC. Therefore, it is advisable to adopt a combination antibiotic regimen if ORC is used.

The results from this study and the studies noted above underscore the importance of selecting appropriate HAs in gynecologic surgery. The significant association between ORC and pelvic abscess formation found in this study suggests that its use in gynecologic procedures should be made with careful consideration, particularly in patients at higher risk of infection or those undergoing extensive surgical interventions. Preoperative antibiotic coverage and postoperative monitoring should be optimized to mitigate these risks. However, the exclusive use of FS in the author’s practice led to the elimination of pelvic abscesses, indicating that FS may be preferable in similar clinical settings. A physiologic clot is formed by FS through the final step of the coagulation cascade, mimicking natural clotting processes [8]. A clot stabilizer (aprotinin) is also included in the formulation of FS that ensures clot integrity during the critical phases of wound healing.

From a practical standpoint, consideration of product availability and cost is important in surgical decision-making. While FS generally has a higher unit cost than ORC, the potential for reduced postoperative complications and readmissions may offset this difference. The lower rates of pelvic abscess and readmission observed with FS in this study suggest that its use could be cost-effective when broader clinical and economic outcomes are considered. Moreover, both products are widely available in most hospital formularies, allowing selection to be guided by patient risk factors, surgical context, and institutional protocols.

This study highlights the need for further research to explore the mechanisms by which ORC contributes to abscess formation and to confirm these findings in larger, multicenter studies. Future research should also investigate whether enhanced antibiotic prophylaxis can consistently reduce the risk of complications with ORC use.

The strengths of this study include the consistency in surgical technique, as all procedures were performed by a single high-volume surgeon, which reduces variability in outcomes. However, the retrospective nature of the study introduces inherent biases, and the use of data from a single surgeon’s practice may limit the generalizability of the findings. Additionally, the relatively small sample size may reduce the power to detect smaller differences in outcomes between the groups. Moreover, the lack of race and ethnicity data, as well as antibiotic regimen variability, may introduce potential confounding factors. As with all single-institution studies, these findings may not be widely applicable to different healthcare settings or geographic regions.

## Conclusions

In conclusion, the use of ORC for adjunctive hemostasis in gynecologic surgery is associated with a significantly higher risk of pelvic abscess formation compared with FS. Surgeons should carefully weigh the benefits against the potential risks when considering the use of ORC, as FS appears to offer a safer alternative for adjunctive hemostasis. Further prospective, multicenter validation is needed to confirm these findings before firm recommendations can be provided.
